# Trading mental and physical health in vestibular schwannoma treatment decision

**DOI:** 10.3389/fonc.2023.1152833

**Published:** 2023-06-26

**Authors:** Kathrin Machetanz, Larissa Lee, Sophie S. Wang, Marcos Tatagiba, Georgios Naros

**Affiliations:** Neurosurgical Clinic, Department of Neurosurgery and Neurotechnology, Eberhard Karls University, Tuebingen, Germany

**Keywords:** vestibular schwannoma (VS), quality of life, mental health, physical health, extent of resection (EOR)

## Abstract

**Objective:**

Observation, radiotherapy and surgery are treatment options in vestibular schwannomas (VS). Decision making differs between centers and is usually based on tumor characteristics (e.g., size) and the expected physical health (PH) outcome (i.e., hearing and facial function). However, mental health (MH) is often under-reported. The objective of the present study was to ascertain the impact of VS treatment on PH and MH.

**Methods:**

PH and MH were assessed in a prospective cross-sectional study including 226 patients with unilateral sporadic VS before and after surgical removal (SURG). Quality-of-life (QoL) was estimated by self-rating questionnaires: general Short-Form Health Survey (SF-36), Penn Acoustic Neuroma Quality-of-Life Scale (PANQOL), Dizziness Handicap Inventory (DHI), Hearing Handicap Inventory (HHI), Tinnitus Handicap Inventory (THI), and Facial Disability Index (FDI). QoL changes over time as well as predictive factors were accessed by multivariate analyses of covariance (MANCOVA).

**Results:**

In total, 173 preoperative and 80 postoperative questionnaires were analyzed. There was a significant PH deterioration related to facial function (FDI, PANQOL-face) after surgery. In line with facial rehabilitation, however, FDI improved within the first five years after surgery and did not differ compared to the preoperative patient cohort, eventually. In contrast, MH (i.e., PANQOL-anxiety) and general health (i.e., PANQOL-GH) improved with surgery and correlated with the extent-of-resection.

**Conclusion:**

Physical and mental health is significantly influenced by VS surgery. While PH might decrease after surgery, MH potentially increases when patient is cured. Practitioners should take MH into account before advising an incompletely VS treatment (e.g., subtotal resection, observation or radiosurgery).

## Introduction

Vestibular schwannomas (VS) are characterized by a progressive loss of cranial nerve (CN) functions (e.g., hearing, balance), affecting patient’s quality of life (QoL) (1–3). Total surgical removal of the tumor is usually providing a definite cure (4, 5). Concurrently, VS surgery implies an increased risk of additional harm to the CN (e.g., facial palsy) (5–10). Observation or radiosurgery are further treatment strategies and in recent years complete VS resection has been discouraged in large VS (11). Instead, current guidelines recommend partial resection (PR) with subsequent radiotherapy in these cases (11). The underlying rationale for this recommendation is to preserve CN function and QoL assuming a linear relationship between them. In fact, several studies report a deterioration of QoL by VS surgery (12–14) relating to hearing, vestibular and facial function (6–8). Radiosurgery or observation has been suggested to affect CN function and QoL to a lesser extent. However, there is increasing evidence that neither radiosurgery nor observation can preserve CN function (in particular hearing) on a long-term (2, 3, 15, 16). Furthermore, some symptoms might be accentuated in comparison to microsurgery (15, 16). Recent studies do not detect any QoL differences when comparing patients following different treatment strategies (14, 17, 18). However, most studies mainly relate to physical health (PH) aspects of QoL. Mental health (MH) referring to the emotional and psychological well-being is often under-reported (17, 19).

An important feature of treatment strategies avoiding a complete VS resection is that it turns a potentially curable disease into a chronic disease with a higher risk of recurrence. It is well known that chronic diseases (e.g., Parkinson’s, cancer) affect patient’s MH independent of their PH (20, 21). In line, two recent studies demonstrated that a gross total resection (GTR) in VS is associated with a better MH compared to partial VS resection (PR) (19). It has been hypothesized that microsurgery may confer an advantage with regard to patient’s MH, relating to the psychological benefit of “cure” from tumor removal (17). In general, however, QoL data in VS treatment differentiating between PH and MH is scarce (14, 17, 18, 22).

The present study aims to investigate the physical and mental health-related QoL in patients with non-treated (before surgery), incompletely (subtotal resection, STR) and completely treated VS (GTR).

## Methods

### Patient characteristics

**Figure 1:**
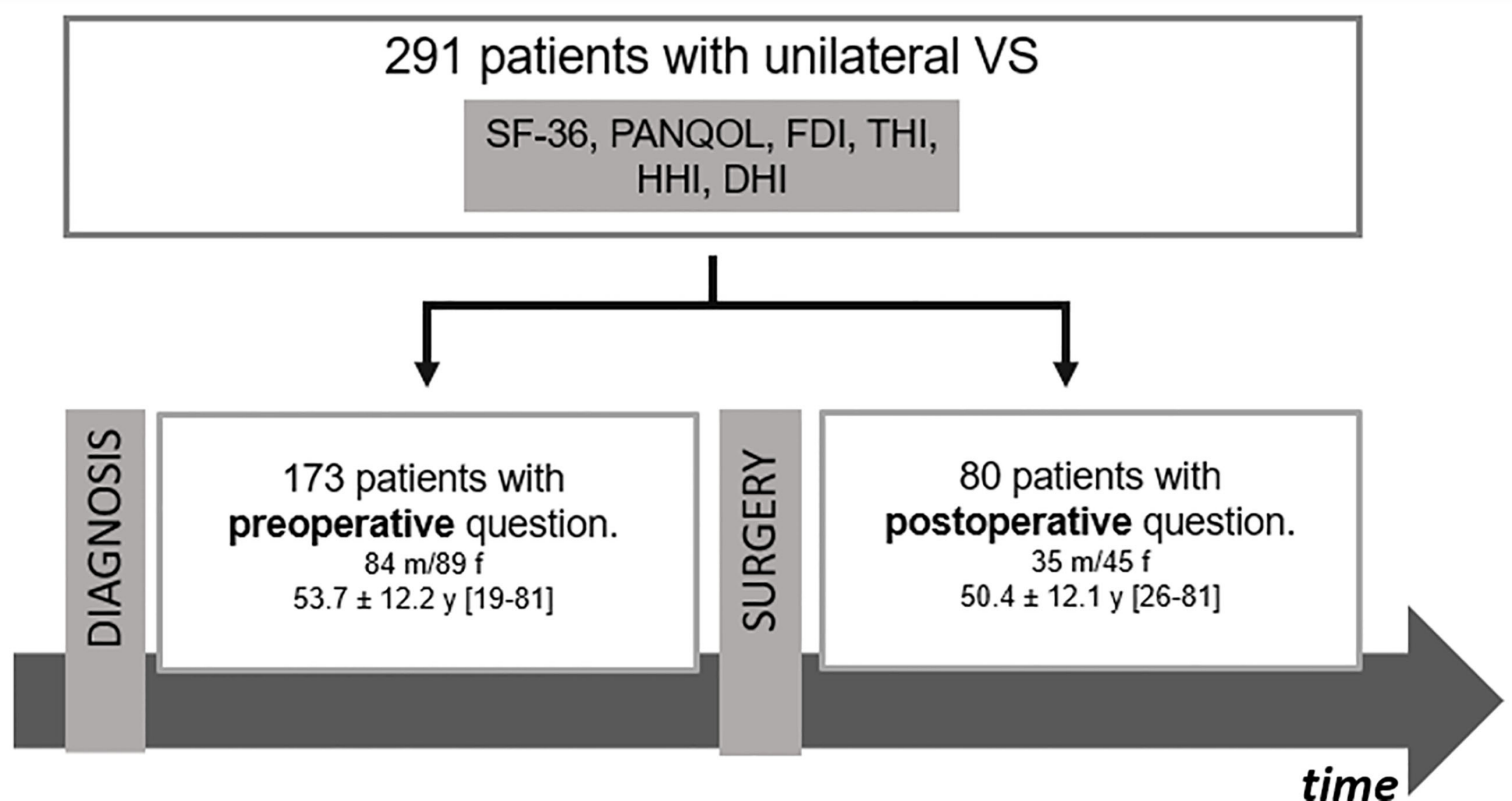
Flow chart of patients’ cohort. DHI, Dizziness Handicap Inventory; FDI, Facial Disability Index; HHI, Hearing Handicap Inventory; PANQOL, Penn Acoustic Neuroma Quality-of-Life Scale; SF-36: Short-Form Health Survey 36.

This prospective cross-sectional study included 226 patients (**Table 1**) with an unilateral VS who answered standardized questionnaires on QoL during their treatment at our Neurosurgical Department between 11/2019 and 09/2021. A total of 141/226 (62.5%) underwent surgical resection of the VS *via* a retrosigmoidal approach in a semi-sitting or supine position in that period (**Figure 1**). Patients with neurofibromatosis, previous VS surgery and incomplete questionnaires were excluded. The study was approved by the local Hospital Ethics Committee and conducted in accordance with the declaration of Helsinki.

**Table 1:** Patient characteristics.

	total	Preoperative	Postoperative	
	n=226 patients	n=173 question.	n=80 question.	
**Gender** *male/* *female*	103/123 (46.6%/54.45%)	84/89 (48.6%/51.4%)	35/45 (43.85%/56.3%)	X^2 = ^0.507 p=0.476
**Age**	53.2 ± 12.4	53.7 ± 12.2	50.4 ± 12.1	p=0.092
**Koos** *T1* *T2* *T3* *T4*	35 (15.5%) 68 (30.1%) 73 (32.3%) 50 (22.2%)	35 (20.2%) 59 (34.1%) 55 (31.8%) 24 (13.8%)	1 (1.3%) 14 (17.5%) 32 (40%) 33 (41.3%)	H=37.99 p<0.001*
**Side** Left/ Right	122/104 (54%/46%)	99/74 (57.2%/42.8%)	36/44 (45%/55%)	X^2 = ^3.29 p=0.07
**Operation** Yes No	141 (62.4%) 85 (37.6%)	88 (50.9%) 85 (49.1%)	80 (100%) 0 (0%)	X^2 = ^59.19 p<0.001*
**Extent of resection** GTR STR PR			56 (70%) 19 (23.7%) 5 (6.3%)	
**H&B** I II III IV V		170 (98.3%) 3 (1.7%)	45 (56.3%) 17 (21.3%) 9 (11.3%) 7 (8.8%) 2 (2.5%)	H=76.2 p<0.001*
**TSD/TSS**		1.28 ± 2.2 y	2.16 ± 3 y	
**SF36** *physical function* *role physical* *bodily pain* *general health* *vitality* *social function* *role emotional* *mental health*		84.6 ± 22.2 69.9 ± 40.0 71.8 ± 29.9 61.1 ± 18.1 55.6 ± 21.3 75.0 ± 25.7 71.5 ± 40.3 68.0 ± 18.2	83.6 ± 17.9 65.3 ± 38.9 76.0 ± 29.3 65.1 ± 19.5 57.3 ± 21.0 77.3 ± 24.0 78.3 ± 37.5 73.3 ± 37.5	p=0.073 p=0.206 p=0.273 p=0.137 p=0.563 p=0.679 p=0.121 p=0.028*
**PANQOL** *anxiety* *facial* *general health* *balance* *hearing* *energy* *pain* *Total*		66.1 ± 22.5 89.5 ± 14.6 55.0 ± 17.3 70.9 ± 24.8 64.0 ± 22.0 67.5 ± 23.7 66.9 ± 29.2 68.6 ± 15.4	73.9 ± 21.8 75.2 ± 23.8 63.9 ± 19.5 67.4 ± 22.5 60.1 ± 22.4 68.1 ± 23.9 70.4 ± 31.9 68.2 ± 16.9	p=0.011* p<0.001* p=0.001* p=0.150 p=0.215 p=0.821 p=0.238 p=0.89
**DHI**		14.4 ± 20.1	19.1 ± 20.9	p=0.032*
**THI**		20.6 ± 21.4	18.2 ± 20.9	p=0.266
**HHI**		20.5 ± 22.4	28.6 ± 22.0	p=0.001*
**FDI** *physical function* *social function*		98.8 ± 8.40 97.7 ± 11.2	86.8 ± 17.6 86.9 ± 17.9	p<0.001* p<0.001*

*bold-marked p-values indicate significance comparing pre- and postoperative patients by a Chi-square (X2) or Kruskal-Wallis test (H).

### Quality of life questionnaires

Several QoL questionnaires were completed during the treatment period: the general Short-Form Health Survey (SF-36), Penn Acoustic Neuroma Quality-of-Life Scale (PANQOL), Dizziness Handicap Inventory (DHI), Hearing Handicap Inventory (HHI), Tinnitus Handicap Inventory (THI), and Facial Disability Index (FDI) (**Table 2**) (23–25).

**Table 2:** General health, disease- and symptom-specific QoL questionnaires.

	Name	Abbr.	Description	No. of items	Best/ worst value	MCID
**SF36 **	Physical: Physical function Role physical Bodily pain General health Mental: Vitality Social functioning Role-emotional Mental health (Reported health transition)	SF36-PF SF36-RP SF36-BP SF36-GH SF36-VT SF36-SF SF36-RE SF36-MH	- Extent to which the health condition affects physical activities such as self-care, walking, climbing stairs, lifting - Extent to which the health condition affects work or other daily activities, e.g. being able to do less than usual, limitations in the type of activities, or difficulty in performing certain activities - Level of pain and impact of pain on normal work - Personal health assessment, including current health status and resistance to illness - Feeling energetic and full of drive vs. tired and exhausted - Extent to which physical health or emotional problems interfere with normal social activities - Extent to which emotional problems interfere with work, or other daily activities, including spending less time, getting less done and not working as diligently as usual - General mental health, including depression, anxiety emotional and behavioral control, general positive mood - Assessment of expected health transition	10 4 2 5 4 2 3 5 1	100/0	8 7
**PANQOL**	Physical: Facial function Balance Hearing Pain Mental: Anxiety Energy General health	PAN-FACE PAN-BAL PAN-HEAR PAN-PAIN PAN-ANX PAN-ENGY PAN-GH	- Level of facial weakness and dysfunction - Level of balance and dizziness complaints - Level of hearing problems - Impact of headache on health related quality of life - Level of anxiety and pain due to the VS - Level of energy, vitality and the ability to concentrate - Assessment of general health and expected health transition	3 6 4 1 4 6 2	100/0	10 16 (14-19) 6 (5-8) 11 (10-13) 11 (5-22) 13 (10-17) 15 (11-19)
**HI**	Hearing HI Tinnitus HI Dizziness HI	HHI THI DHI	- Assessment for self-perceived hearing handicap - Assessment for self-perceived tinnitus handicap - Assessment for self-perceived dizziness handicap	25 25 25	0/100 0/100 0/100/0	12 7 18
**FDI**	Physical function Social function	FDI-PF FDI-SF	- Level of limitations in physical disability, e.g. problems with instrumental activities of daily living and difficulty with producing appropriate facial expressions - Social and emotional problems experienced due to facial dysfunction	5 5	100	

SF36: 36-item Short-Form Health Survey, PANQOL, Penn Acoustic Neuroma Quality-of-Life Scale; HI, Handicap Inventory; FDI, Facial Disability Inde; literature-based minimal clinically important differences (MCID) [Carlson et al., 2015 (23); Newman et al., 1991 (24); Zeman et al., 20 (25)].

The SF-36 is the most common health-related QoL questionnaire. Its 36 items can be divided into physical and mental classes with 4 domains each: physical function (SF36-PF), role-physical (SF36-RP), bodily pain (SF36-BP), general health (SF36-GH), vitality (SF36-VT), social functioning (SF36-SF), role-emotional (SF36-RE) and mental health (SF36-MH). Each domain is scored from 0-100, with a higher score corresponding to a better QoL.

The PANQOL is a disease-specific questionnaire containing 26 questions which are divided into the domains anxiety (PAN-ANX), facial function (PAN-FACE), general health (PAN-GH), balance (PAN-BAL), hearing (PAN-HEAR), energy (PAN-ENGY) and pain (PAN-PAIN). A total score (PAN-TTL) is calculated from the individual scores. The response options are classified on a Likert scale from strong disagreement (1) to strong agreement (5), whereby the values are normalized to a scale of 0-100 points to determine the domain scores. A score of 100 corresponds to the best possible QoL, a score of 0 to the lowest QoL.

The HHI, THI and DHI are symptom-specific questionnaires for dizziness, hearing function and tinnitus. Each questionnaire contains 25 self-assessment items, which can be answered by yes (2), sometimes (1) or no (0). Item scores result in a total score of 0-100, whereby a higher score corresponds to greater impairment by the symptom. The FDI were administered to all patients with facial paresis. The FDI contains 10 questions, which are divided into the domains *physical function* (-25=worst to 100=best function; FDI-PF) and *social function* (0=worst to 100=best function; FDI-SF).

### Disease-specific data

In addition, we analyzed numerous disease specific information. Magnetic resonance images (MRI) were retrospectively analyzed to determine the tumor size according to Koos classification (1: purely intrameatal, 2: intra- and extrameatal, 3: filling the cerebellopontine cistern, 4: compressing or shifting the brainstem) (26) and tumor side. The extent of resection (EOR) after surgery (GTR: complete resection; STR: minimal residual tumor on the facial nerve or exclusively in the internal auditory canal; PR: great tumor volume) was determined by MRI and the surgical record. Medical records of patients were reviewed to define the time between diagnosis and QoL survey (time since diagnosis, TSD; preoperatively), time between preoperative QoL survey and surgery (time before surgery, TBS; preoperatively; only patients who answered the preoperative survey and underwent VS resection at a later time during the evaluation period), time between surgery and postoperative survey (time since surgery, TSS; postoperatively) as well as the facial function according to the House-Brackmann scale (H&B) (27). The H&B classifies overall facial function into ranges from 1 (normal) to 6 (total paralysis) based on the assessment of e.g. eye closure and mouth movement.

### Statistics

Statistical tests were performed using SPSS (IBM SPSS Statistics for Windows, Version 26.0. Armonk, NY: IBM Corp.). Group differences in distribution of clinical characteristics (e.g., EOR) were determined by Chi-squared or Kruskal-Wallis tests. In a first step, (multivariate) analyses of covariance ((M)ANCOVAs) were performed to evaluate the effects of surgery (SURG) and gender (SEX) on QoL scores. Secondary, a (M)ANCOVA-based evaluation of the impact of TSD, TSS and EOR on QoL was performed. In order to ensure that results were not influenced by assumption violations, data were checked for outliers, homogeneity of variance–covariance matrices (Box’s M test) and homogeneity of variances (Levene’s test). In this context, a MANCOVA is a two-step process. In the first step, the overall hypothesis is tested, i.e. whether there is a difference between different groups. If this test is significant, in the second step the MANCOVA was followed by *post-hoc* tests (i.e. univariate ANOVAs) to explain the group differences. Furthermore, we performed a secondary subcohort analysis of patients who completed the questionnaires in both the pre- and postoperative period. To estimate differences in QoL scores before and after surgery we performed a repeated measures ANOVA. Statistical significance was considered at p < 0.05 for all statistical tests.

## Results

### Patient cohort

A total of 226 patients (53.2 ± 12.4 years; 123 female) completed all QoL questionnaires. 103/226 (45.6%) of the VS corresponded to a tumor size Koos 1/2 and 123/226 (54.4%) to a grade 3/4 (**Table 1**). VS were resected in 141/226 (62.4%) of the patients, while 85/226 (37.6%) had not undergone surgery at the time of evaluation (**Figure 1**). In summary, 27/226 (11.9%) patients completed questionnaires pre- and postoperatively (**Supplementary Table 1**), whereas 146/226 (64.6%) and 53/226 (23.5%) were surveyed only pre- or postoperatively, respectively.

### Common health-related QoL: SF36

**Figure 2:**
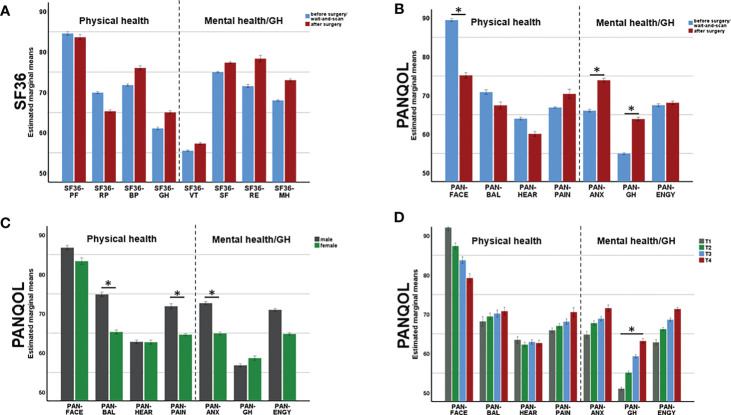
Changes of physical (PH) mental health (MH) after surgery. While SF36 **(A)** did not depict any surgery-related changes in QoL, PANQOL **(B)** showed a decline of PH (related to the facial function, PAN-FACE) after surgery. At the same time, surgery improved MH related to anxiety (PAN-ANX) and general health (PAN-GH). Multivariate analysis also depicted an effect of gender **(C)** and tumor size **(D)** on QoL. Bars in **(C, D)** demonstrate data from both, pre- and postoperatively. PANQOL, Penn Acoustic Neuroma Quality-of-Life Scale; PAN-ANX, PANQOL anxiety; PAN-ENGY, PANQOL energy; PANQOL-GH, PANQOL general health; PAN-FACE, PANQOL facial; PAN-BAL, PANQOL balance; PAN-HEAR, PANQOL hearing; PAN-PAIN, PANQOL pain; SF36-PF: SF36 physical function; SF36-RP: SF36 role physical; SF36-BP: SF36 bodily pain; SF36-GH: SF36 general health; SF36-VT: SF36 vitality; SF36-SF: SF36 social functioning; SF36-RE: SF36 role emotional; SF36-MH: SF36 mental health. Significance is indicated by an asterisk (*; p<0.05, MANOVA).

A MANCOVA was applied to SF36 subdomains in order to determine the effect of surgery (SURG) on QoL while controlling for SEX, AGE and tumor size (SIZE) (**Figure 2A**). Neither SURG (F_(8,240)_=1.09, p=0.374) nor SEX (F_(8,240)_=1.37, p=0.21) had a significance effect on QoL. In contrast, MANOVA depicted a significant effect of AGE on SF36 (F_(8,240)_=3.72, p<0.001). Follow-up ANOVAs confirmed a significant impact of AGE on PH as depicted by SF36-PF (F_(1,247)_=12.74, p<0.001) and SF36-GH (F_(1,247)_=5.50, p=0.020). Independent of the other covariates, PH items (SF36-PF and SF36-GH) decreased with age (r =-0.2, p=0.001 and r=-0.15, p=0.016).

### Disease-specific QoL: PANQOL

**Figure 3:**
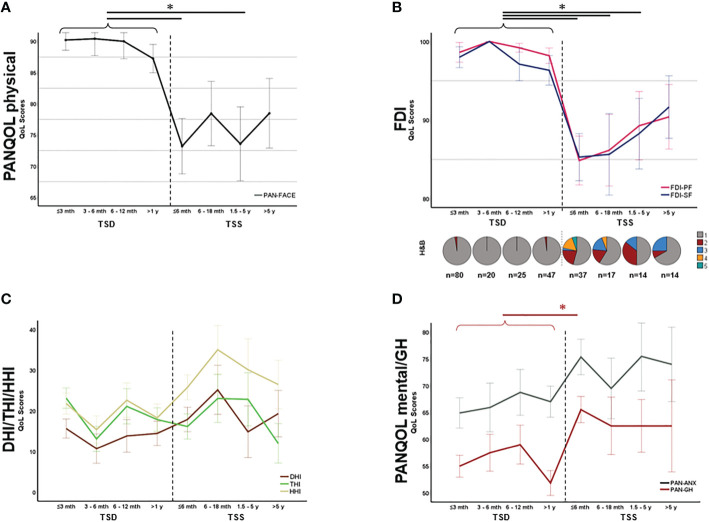
Symptom-specific quality of life (QoL) over time. PAN-FACE **(A)** and FDI scores **(B)** improved after surgery in line with the facial rehabilitation (see distribution of H&B scores in the inlay). After 5 years, PAN-FACE and FDI differed significant in comparison to the preoperative patient cohort. In contrast, neither DHI, THI and HHI **(C)** nor PAN-ANX/PAN-GH **(D)** changed during follow-up. The numbers under the pie charts in **(B)** indicate the total number of patients in each time period. DHI, dizziness handicap inventory; THI, tinnitus handicap inventory; HHI, hearing handicap inventory; FDI-PF, Facial disability index - physical function; FDI-SF, Facial disability index – social function; TSD, time-since-diagnosis; TSS, time-since-surgery. Significance is highlighted by an asterisk (*; p<0.05, Dunn’s test, corrected).

A MANCOVA was performed to estimate the effect of SURG on PANQOL subdomains while controlling for SEX, AGE and SIZE. There was a significant multivariate main effect of SURG (F_(7,241)_=7.75, p<0.001; **Figure 2B**). SURG improved mental and general health in the subdomains PAN-GH (63.9 ± 19.5 vs 55.0 ± 17.3; F_(1,247)_=4.86, p=0.028) and PAN-ANX (73.9 ± 21.8 vs 66.1 ± 22.5; F_(1,247)_=5.68, p=0.018). In contrast, PH relating to facial function (PAN-FACE) decreased postoperatively on a group level (75.2 ± 23.8 and 89.5 ± 14.6; F_(1,247)_=27.35, p<0.001) (**Figure 3A**).

MANCOVA also proved a significant main effect of SEX on PANQOL (F_(7,241)_=3.49, p=0.001; **Figure 2C**). Females had significant worse PAN-ANX (64.9 ± 22.0 vs 72.6 ± 22.5; F_(1,247)_=7.44, p=0.007), PAN-BAL (65.3 ± 24.8 vs 74.9 ± 22.4; F_(1,247)_=9.47, p=0.002) and PAN-PAIN (64.6 ± 31.3 vs 71.9 ± 28.1; F_(1, 247)_=7.56, p=0.006) scores independent of the actual VS treatment. Furthermore, both covariates AGE (F_(7,241)_=5.93, p<0.001) and SIZE (F_(7,241)_=2.13, p=0.04) had significant impact on PANQOL. Increasing AGE had a negative effect on facial function (PAN-FACE; r=-0.18, p=0.005; Spearman’s), balance (PAN-BAL; r= -0.15, p=0.001; Spearman’s) and hearing (PAN-HEAR; r=-0.15, p=0.016; Spearman’s). PAN-GH was significantly impacted by the SIZE (F_(1,247)_=5.47, p = 0.02) with better values in Koos 3/4 compared to Koos 1/2 tumors (H=14.75, p=0.001; Kruskal-Wallis) (**Figure 2D**).

### Symptom-specific QoL: HHI, THI, DHI and FDI

In the MANCOVA, there was a significant effect of SURG (F_(3,245)_=4.96, p=0.002), SEX (F_(3,245)_=7.55, p<0.001) and AGE (F_(3,245)_=3.94, p=0.009) on the handicap inventories (i.e., DHI, HHI and THI). In the follow-up ANOVAs, both SURG (20.5 ± 22.4 vs 28.6 ± 22.0; F_(1,247)_=7.55, p=0.006) and AGE (F_(1,247)_ =3.89, p=0.05) had negative impact on hearing perception (HHI). In contrast, females suffered from dizziness (DHI) more frequently than males regardless of VS treatment (20.4 ± 22.4 and 10.8 ± 16.6; F_(1,247)_=13.47, p<0.001). Tinnitus perception (THI) was unaffected by SURG, SEX or AGE in the present cohort.

Both FDI subscores representing the physical (FDI-PH) and social handicap (FDI-SH) of a facial palsy were negatively affected by SURG (F_(1,247)_=36.9, p<0.001 and F_(1,247)_=23.25, p<0.001). The covariate SIZE had impact only on FDI-PH (F_(1, 247)_ = 4.51, p=0.035). FDI-PF and FDI-SF correlated significantly with H&B (r=-0.88, p<0.001 and r=-0.85, p<0.001; Spearman’s.

### Impact of time since diagnosis, time before/since surgery as and extent of resection on patients’ QoL

In order to evaluate the impact of timing of the survey after VS diagnosis and before surgery on mental health, the association between PAN-GH and PAN-ANX as well as TSD and TBS was analyzed. Among the 173 preoperative questionnaires, there was no significant correlation between TSD and PAN-ANX or PAN-GH. Furthermore, in the 88/173 patients who completed a questionnaire in the observation phase and underwent surgery later during the evaluation period, no correlation between TBS and mental health was found either (PAN-ANX: r=0.021, p=0.843; PAN-GH: r=-0.18, p= 0.093; Spearman’s).

**Figure 4:**
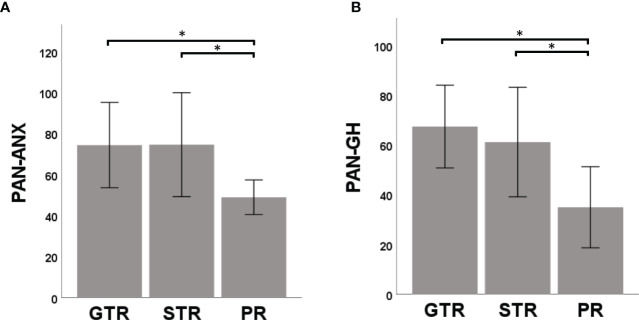
Relationship between mental health and extent-of-resection (EOR). Both PAN-ANX **(A)** and PAN-GH **(B)** correlated with EOR. Patients with significant residual tumor (partial resection, PR) claimed higher level of anxiety and a reduced level of general health in comparison to patients undergoing a gross total resection (GTR) or subtotal resection (STR). Significance is highlighted by an asterisk (*; p<0.05, Dunn’s test, corrected).

As the functional status after surgery is constantly changing due to rehabilitation mechanisms, we ought to evaluate health-related QoL depending on the TSS. In fact, for both FDI-PF (H=56.65, p<0.001; Kruskal Wallis) and FDI-SF(H=53.93, p<0.001; Kruskal Wallis) there was a significant decline of QoL directly after surgery which improved during the postoperative course in line with facial rehabilitation (**Figure 3B**). After a TSS of approx. 5 years, there was no significant difference in neither FDI-PF (H=36.16, p=0.122; Kruskal Wallis) nor FDI-SF (H=33.17, p=0.240; Kruskal Wallis) when compared to the preoperative situation (**Figure 3B**). There was no comparable effect for the disability inventories THI, HHI, DHI or PAN-ANX and PAN-GH (**Figures 3C, D**). In contrast, postoperative mental and general health parameters (PAN-ANX and PAN-GH) were associated with the EOR. Kruskal-Wallis test revealed a significant better PAN-ANX (H=6.81, p=0.033) and PAN-GH (H=10.63, p=0.005) in GTR and STR in comparison to PR (**Figures 4A, B**).

## Discussion

The present study evaluated main determinants of physical and mental health in patients with VS. While PH and MH did not change after diagnosis, deterioration of PH was detected postoperatively - mainly caused by an occurrence of facial nerve palsy and the deterioration of hearing function. However, PH related to facial function improved within the first years after surgery. Furthermore, mental and general health improved postoperatively and correlated with the EOR. The decision on therapy is therefore a consideration between MH and PH and must be made on a patient-specific basis.

Current guidelines for VS advise observation or radiotherapy and discourage complete VS resection to preserve CN function (11). The present study documents a significant postoperatively deterioration of facial and hearing QoL scores with a similar or even less pronounced extent compared to previous studies (13, 28). The retrosigmoid approach in this context may have resulted in less hearing loss compared to studies applying a translabyrinthine approach. However, physical limitations should not only be compared pre- and postoperatively, but also functional recovery after surgery should be considered when deciding on treatment. Our findings elicit an improvement of FDI-PF and FDI-SF over time after microsurgery. Nevertheless, our study could not detect a significant effect of TSS alone on facial function. This could be attributed to a data bias, since patients without physical complaints usually no longer present themselves in our outpatient clinic after approx. 3 years. Thus, an overrepresentation of patients with impairing facial palsy must be assumed in our postoperative cohort. In fact, previous studies show heterogenous results regarding longitudinal facial palsy-specific QoL (29, 30). Further longitudinal studies are necessary to assess the frequency, course of recovery and subjective limitation of facial palsies after VS resection.

Tinnitus and dizziness are symptoms often associated with VS. Nevertheless, they are often underrated when deciding on the treatment strategy. Tinnitus and vertigo, however, can significantly worsen QoL in VS patients (23, 31). Thus, we determined tinnitus-related QoL by the THI. Tinnitus-related discomfort tended to improve slightly although previous reported “minimal clinically important difference” (MCID) could not be reached (25). This is concordant with previous studies demonstrating postoperative improvement in patients with preoperative tinnitus, while patients without preoperative tinnitus can develop a new-onset tinnitus after surgery in ~20% (32–34). Consequently, patients with preoperative severe tinnitus could be offered microsurgical resection of the VS, as radiotherapy may worsen tinnitus-related discomfort (35). The results of studies investigating pre- and postoperative dizziness in VS are ambiguous (36). Our study could not reveal pre- and postoperative differences of DHI and PAN-BAL. Instead, more dizziness was associated with female gender and higher age.

The relevance of MH on overall health is often underestimated in the treatment of benign tumors. While there are numerous studies on MH in meningiomas (37–39), data on MH in VS are scarce. The present study could not demonstrate an effect of surgery on mental or physical SF-36 scores, confirming the assumption about low predictability of QoL in VS by the SF-36 (40, 41). However, despite the deterioration in PH the PANQOL findings demonstrated a significant increase of mental and general health post-surgically. In contrast, during the preoperative observational phase there was a deterioration of mental scores over time. This suggests that patients experience relief from treatment, whereas knowledge of the presence of a VS without treatment leads to a state of anxiety. These result supports the hypothesis of Carlson et al. which suggests that microsurgery may improve patient’s MH when the tumor is “cured” after complete surgical removal (17). While previous studies could demonstrate a reduced QoL of VS patients in comparison to age and sex matched normative data already before surgery, conversely, they could not prove a significant difference of MH between observational, microsurgery and radiotherapy groups (14, 17, 18, 28, 42–44). However, factors affecting the results (e.g., EOR, gender) are not taken into account in these studies. While multivariate statistics could not demonstrate an effect of EOR on MH scores, univariate analysis demonstrated a significantly worse PAN-GH in partial resections compared to STR and GTR. This is concordant with studies comparing GTR with incomplete resection or combined radio- and microsurgery (19, 28). Since other studies furthermore demonstrated a significant regrowth rate with a tumor residue of >0.7 cm^3^ and a higher MIB-1 index (45, 46), general recommendation for PR in large VS should be avoided. Instead, multicenter studies, that prospectively assign patients to different intention-to-treat groups (i.e. intended GTR, intended PR), are necessary.

### Limitations

The present study is limited due to the lack of comparison with radiosurgery or other surgical procedures (e.g., translabyrinthine surgery), a non-tumor cohort and the absence of longitudinality. The small number of postoperative controls can lead to a selection bias of the health status, since at long-term, patients with persistent complaints continue to present themselves in the consultation, while patients with good health no longer present themselves.

## Conclusion

In VS patients, the trading of MH and PH is essential for treatment decision making. While mental health in particular is impaired preoperatively, patients are impaired postoperatively, especially due to physical problems related to cranial nerve dysfunction. Attending physicians should take this into account during treatment decision making.

## Data availability statement

The raw data that support the findings of this study are available from the corresponding author upon reasonable request.

## Ethics statement

The studies involving human participants were reviewed and approved by Ethics committee of the medical faculty of Eberhard Karls University Tübingen and conducted in accordance with the declaration of Helsinki.

## Author contributions

Study conception and design: KM, GN. Data acquisition: KM, LL, SW. Data analysis: GN, KM. Data interpretation: KM, SW, MT, GN. Statistical analysis: KM, GN. writing of the first draft: KM, GN. Review of the final manuscript: KM, MT, GN. All authors contributed to the article and approved the submitted version.

## Supplementary material

Supplementary Table 1 QoL values of patients who completed the questionnaires both in the pre- and postoperative period. *p-values indicate significance pre-and postoperatively by a repeated measures ANOVA.
