# Supplementary material for: Trading mental and physical health in vestibular schwannoma treatment decision
Source: Front Oncol. 2023 Jun 26;13:1152833. doi: 10.3389/fonc.2023.1152833 (PMC10332305; doi:10.3389/fonc.2023.1152833)
Supplement: Supplementary Table 1 — QoL values of patients who completed the questionnaires both in the pre- and postoperative period. *p-values indicate significance pre-and postoperatively by a repeated measures ANOVA. [file Table_1.pdf]

	**Preoperative** n=27	**Postoperative** n=27	
**HB**			
I	25 (92.6%)	15 (55.6%)	
II	2 (7.4%)	8 (29.6%)	
III	0 (0%)	0 (0%)	
IV	0 (0%)	3 (11.1%)	
V	0 (0%)	1 (3.7%)	
**SF36**			
physical function	94.1 ± 8.2	85.0 ± 14.9	**p=0.009***
role physical	75.0 ± 38.0	60.2 ± 36.9	p=0.080
bodily pain	81.0 ± 24.7	82.1 ± 24.9	p=0.765
general health	71.0 ± 15.7	66.4 ± 18.8	p=0.278
vitality	63.3 ± 19.6	54.3 ± 19.7	**p=0.029***
social function	79.2 ± 21.9	75.0 ± 22.2	p=0.420
role emotional	86.4 ± 29.6	88.9 ± 24.5	p=0.703
mental health	75.9 ± 14.6	76.4 ± 16.2	p=0.827
**PANQOL**			
anxiety	71.5 ± 21.9	75.5 ± 21.3	p=0.205
facial	92.6 ± 12.1	78.7 ± 23.8	**p=0.007***
general health	62.0 ± 19.1	64.4 ± 16.9	p=0.551
balance	79.6 ± 18.0	69.3 ± 21.9	p=0.060
hearing	70.6 ± 21.6	63.7 ± 19.5	p=0.150
energy	78.5 ± 19.0	69.9 ± 19.7	**p=0.030***
pain	75.9 ± 25.5	78.7 ± 27.5	p=0.558
Total	75.8 ± 12.8	71.4 ± 14.0	p=0.099
**DHI**	11.0 ± 13.1	19.9 ± 19.0	**p=0.019***
**THI**	12.4 ± 12.6	16.9 ± 20.5	p=0.214
**HHI**	14.5 ± 14.9	24.9 ± 19.0	**p=0.004***
**FDI**			
physical function	95.6 ± 19.2	88.2 ± 15.6	p=0.152
social function	94.1 ± 20.1	87.0 ± 16.1	p=0.155

Supplementary Table 1: QoL values of patients who completed the questionnaires both in the pre- and postoperative period.
*p-values indicate significance pre-and postoperatively by a repeated measures ANOVA
